# Supplementary material for: Rationale and design of the brain magnetic resonance imaging protocol for FutureMS: a longitudinal multi-centre study of newly diagnosed patients with relapsing-remitting multiple sclerosis in Scotland
Source: Wellcome Open Res. 2022 Mar 16;7:94. [Version 1] doi: 10.12688/wellcomeopenres.17731.1 (PMC9971644; doi:10.12688/wellcomeopenres.17731.1)
Supplement: Peer review history

# Peer review: Reviewer response for version 1

**DOI:** 10.21956/wellcomeopenres.19621.r54070

**Published:** 2023-01-30

**Authors:** Renske Gahrmann

**Affiliations:** 1 Department of Radiology and Nuclear Medicine, Erasmus MC University Medical Centre Rotterdam, Rotterdam, The Netherlands

The authors describe a comprehensive overview of all imaging protocols performed in different hospitals in the FutureMS study. The study itself and the radiological analysis seems very ambitious.

 Postprocessing methods are described in extensive detail for future study reference. Background of different structural and functional imaging techniques as well as the rationale behind using these techniques for the evaluation of MS-related changes are provided. The simultaneous evaluation of so many different parameters will hopefully lead to more comprehensive relatable findings, especially compared to most smaller studies that seem to focus on a single parameter only.

*Comment1*:

 The authors state two main primary study endpoints based on imaging findings: new/enlarging WML and brain volume reduction over 1 year. There are also secondary endpoints based on advanced MRI technique-outcomes. The description of these measures as ‘outcomes’ seems a bit confusing as not all patients will have changes on MRI after 1 year and the study continues for several years with clinical checks. Should the radiological results not be related to the clinical outcome instead of making the radiological results a separate outcome measure?

*Comment2*:

 In the Introduction, under Structural MRI in MS, the authors state that cortical brain lesions are difficult to detect on 1.5T and 3T MRI. But this tends to be very sequence-dependent. On Double-Inversion Recovery sequences for instance, cortical lesions are often apparent.

*Comment3*:

 Table1 baseline demographics seems very limited to the point where it is questionable whether a table is at all necessary.

*Comment4*:

 The extensive study protocol only includes non-contrast sequences. Breakdown or dysfunction of the BBB is a feature of active inflammatory MS plaques. Rim lesions on SWI are postulated to represent chronic inflammatory plaques, but not acute inflammation. Could the presence of active plaques affect the structural and functional results? Is there another way to measure plaque activity using the currently chosen sequences?

*Comment5*:

 There are 16 tracts of interest chosen for quantitative tractography. Why were these tracts and this number of tracts chosen? Are there sources stating that these tracts are most likely affected in MS?

Is the study design appropriate for the research question?

Yes

Is the rationale for, and objectives of, the study clearly described?

Yes

Are sufficient details of the methods provided to allow replication by others?

Yes

Are the datasets clearly presented in a useable and accessible format?

Yes

Reviewer Expertise:

I am a neuroradiologist with a PhD in MR-imaging in neuro-oncology. My MS experience is clinical.

I confirm that I have read this submission and believe that I have an appropriate level of expertise to confirm that it is of an acceptable scientific standard.

# Peer review: Reviewer response for version 1

**DOI:** 10.21956/wellcomeopenres.19621.r49263

**Published:** 2022-11-15

**Authors:** Olga Ciccarelli

**Affiliations:** 1 Faculty of Brain Sciences, University College London Queen Square Institute of Neurology, University College London, London, UK

This report describes the MRI acquisition, processing and management of data from patients enrolled into the FutureMS cohort. In additional to conventional sequences, advanced imaging sequences were also acquired. 

 I have a very few and minor comments/suggestions:

- Can the authors report other baseline characteristics? For example, disease duration? type of onset?
- What is the interval between baseline MRI and last relapse?
- Can the number of patients who underwent the sub-study sequences be given in the methods?
- Are any clinical and demographic data available on the follow-up scans?

Is the study design appropriate for the research question?

Yes

Is the rationale for, and objectives of, the study clearly described?

Yes

Are sufficient details of the methods provided to allow replication by others?

Yes

Are the datasets clearly presented in a useable and accessible format?

Yes

Reviewer Expertise:

MS, imaging

I confirm that I have read this submission and believe that I have an appropriate level of expertise to confirm that it is of an acceptable scientific standard.
